# Room-Temperature Synthesis of ZnS Nanoparticles Using Zinc Xanthates as Molecular Precursors

**DOI:** 10.3390/ma13010171

**Published:** 2020-01-01

**Authors:** Neli Mintcheva, Gospodinka Gicheva, Marinela Panayotova, Sergei A. Kulinich

**Affiliations:** 1 Department of Chemistry, University of Mining and Geology, Sofia 1700, Bulgaria; e_gospodinka@yahoo.com (G.G.); marichim@mgu.bg (M.P.); 2 Research Institute of Science and Technology, Tokai University, Hiratsuka, Kanagawa 259-1292, Japan; 3 Department of Mechanical Engineering, Tokai University, Hiratsuka, Kanagawa 259-1292, Japan

**Keywords:** ZnS nanoparticles, zinc xanthate, triethanolamine, photocatalysis

## Abstract

Molecular precursors are suitable starting compounds for preparation of semiconductor nanoparticles (NPs), which allow for control of atomic ratio, composition, monodispersity, and particle size of nanoscaled metal sulfides/oxides. In the present study, we carried out a one-pot synthesis of ZnS NPs in aqueous triethanolamine medium at room temperature, from molecular precursor zinc xanthate as a source of both Zn^2+^ and S^2−^ ions. Furthermore, we compared the products obtained from zinc ethylxanthate (Zn(C_2_H_5_OCS_2_)_2_) and zinc amylxanthate (Zn(C_5_H_11_OCS_2_)_2_). The as-prepared ZnS NPs were found to crystallize in cubic phase, which usually forms at low temperatures, with the dimension dependent on the xanthate precursor used. The long carbon-chain xanthate Zn(C_5_H_11_OCS_2_)_2_ gave spherically shaped NPs with an average diameter of 19 nm, while the NPs that originated from zinc ethylxanthate had a mean size of ~26 nm. Both nanomaterials had surface sulfur vacancies that extended their absorption spectra toward the visible region and reduced the band gap. This allowed both materials to demonstrate photocatalytic performance under visible-light irradiation. Photodegradation of methylene blue over newly prepared ZnS NPs was tested under visible light, demonstrating efficiency of 50%–60% after 180 min.

## 1. Introduction

During the last decades, semiconductor nanoparticles (NPs) have been extensively explored because such nanomaterials often demonstrate unique properties that are attractive for applications in various fields, such as optics, electronics, biomedicine, sensing, photocatalysis, and so on. The distinctive properties of semiconductor nanomaterials are well known and result from their chemical and phase composition, NP size and shape, surface chemistry, and defects. Zinc sulfide, as a type II–VI semiconductor, is among the widely investigated materials, and many methods have been proposed for the preparation of its nanostructures with tailored characteristics. For example, the hydrothermal method is commonly used for synthesis of nanosized ZnS from starting reagents such as sodium sulfide, hydrogen sulfide, thiourea, and sulfur powder [1,2,3,4,5,6]. The NP size and composition can be controlled by changing the pH and/or adding a stabilizing agent [7,8,9,10]. A number of methods have been applied in order to prepare stable and defect-rich nanomaterials with enhanced properties, such as the microwave irradiation technique [11,12], low-temperature microemulsion [13,14], aerosol microprecipitation [15], capping ZnS NPs with polymer [16], or even biological methods like *Stevia*-assisted precipitation [17] and sulfate reduction by leaf extract [18].

Among the chemical methods for preparation of semiconductor NPs, high-temperature thermolysis of a molecular precursor is a useful approach to achieve monodispersed NPs with well-controlled size [19,20,21,22,23,24]. Atomic rearrangement takes place throughout the NP formation process from a single-source compound, resulting in a uniform distribution of metal ions and retention of the initial stoichiometry in the final product. In parallel, a stepwise decomposition of the precursor allows for the control of nucleation and crystal growth of NPs. Moreover, ligands strongly coordinated to the metal-ion center of transition metal complexes may affect the NP formation processes and change the crystal phase of the formed NPs, as previously shown by Cheng et al. [19]. Coordinated organic molecules of the precursor are able to act as in-situ capping agents for created NPs [20]. As an example, Liu et al. reported on the use of glutathione as both ligand and sulfur source for luminescent CdS NPs with tunable color [21]. 

Thermal decomposition of coordination compounds with S-containing ligands (such as dithiocarbamate, dithiocarbonate (xanthate), trithiocarbonate, thiosemicarbazone, and thiobiuret), which serve as sulfide sources, has already been applied to produce metal sulfide NPs with good crystallinity, narrow size distribution, and tunable dimensions [22,23,24,25]. For instance, a hot-injection route, which uses thermolysis of metal (Zn, Cd, Hg) complexes in alkylamine at 230–280 °C, was shown to lead to formation of monodispersed metal sulfide NPs wrapped with amines [26,27,28,29,30]. Interestingly, aggregates of CdS with sizes of 140–200 nm were obtained from complex Cd(ethylxanthate)_2_, while NPs with diameters of 4–7 nm were formed from diamine–ethylxanthate complex of Cd(II), indicating the protective role of strongly coordinated diamine ligand for the metal center [31]. Furthermore, cadmium ethylxanthate has been applied to form CdS NPs under different reaction conditions [32,33,34]. Nair et al. synthesized CdS NPs capped with tri-*n*-octylphosphine oxide (TOPO) through the thermolysis of cadmium ethylxanthate at 280 °C in inert atmosphere [32]. Multipodal CdS nanostructures were formed from the same single precursor in ethylenediamine solution in a temperature range of 100–180 °C [33], while long-chain alkyl xanthates of cadmium dissolved in hexadecylamine were found to give CdS NPs upon heating to temperatures 70–120 °C [34].

It is worth noting that all the above syntheses were carried out at elevated temperatures and typically in inert atmosphere. Pradham et al. reported on the production of metal sulfide crystalline particles with tunable size and shape via metal alkyl xanthates by employing alkylamines as solvents, which were found to play a crucial role in the synthesis, significantly lowering the decomposition temperature of metal xanthate precursors [23]. This finding stimulated us to explore the effect of hydroxyl alkyl amines on the formation of metal sulfide NPs with metal xanthate as a molecular precursor. Therefore, our work aimed to achieve formation of metal sulfide NPs from a xanthate source in ambient conditions and with water as medium. To the best of our knowledge, little information on ZnS NPs obtained from zinc xanthates is available to date [35,36], which is why it is worth exploring how such nanostructures can be controlled under different reaction conditions.

Hence, in this work, we studied the preparation of ZnS NPs from a molecular precursor zinc ethylxanthate (Zn(C_2_H_5_OCS_2_)_2_) or zinc amylxanthate (Zn(C_5_H_11_OCS_2_)_2_) as a source of sulfide and zinc ions in reaction with triethanolamine (N(C_2_H_4_OH)_3_), which acted as both nucleophile and a capping agent at room temperature. We report a facile method for the preparation of ZnS NPs with tunable size depending on the length of the carbon chain in the xanthate molecule used as the sulfide source. Considering the remarkable interest in applying ZnS nanostructures in diverse photocatalytic reactions under visible-light irradiation [37,38], upon characterizing them properly, we then tested the performance of the as-prepared nanomaterials in degradation of methylene blue (MB), which was used as a model compound simulating organic pollutants in wastewaters. 

## 2. Materials and Methods 

### 2.1. Reagents and Materials

Potassium ethylxanthate (C_2_H_5_OCS_2_K; Acros Organics, Geel, Belgium), potassium amylxanthate (C_5_H_11_OCS_2_K; TCI, Tokyo, Japan), Zn(NO_3_)_2_·6H_2_O (Teocom, Sofia, Bulgaria), triethanolamine (N(C_2_H_4_OH)_3_; Teocom, Sofia, Bulgaria), and ethanol (Teocom, Sofia, Bulgaria) were all analytical grade reagents purchased from the corresponding manufacturers and used as supplied and without any further purification. 

### 2.2. Synthesis of Zn(C_2_H_5_OCS_2_)_2_ and Zn(C_5_H_11_OCS_2_)_2_

At room temperature, 0.7437 g (2.50 mmol) of Zn(NO_3_)_2_·6H_2_O dissolved in 10 mL of distilled water was added into 30 mL of aqueous potassium ethylxanthate with 0.8000 g (5.00 mmol) of solute. The obtained precipitate of Zn(C_2_H_5_OCS_2_)_2_ was filtrated, washed with water and ethanol subsequently, and then dried in air. In a similar way, the powder of Zn(C_5_H_11_OCS_2_)_2_ was prepared using potassium amylxanthate (1.012 g (5.0 mmol)). Both rinsed and dried compounds were then used as precursors for one-pot synthesis of ZnS NPs.

### 2.3. Preparation of ZnS Nanoparticles 

In a typical procedure, 0.2156 g (0.70 mmol) of Zn(C_2_H_5_OCS_2_)_2_ or 0.2745 g (0.70 mmol) of Zn(C_5_H_11_OCS_2_)_2_) was added to 6 mL of triethanolamine and 6 mL of distilled water to prepare samples ZnS(1) and ZnS(2), respectively. The reaction mixture was stirred at room temperature for three days until a pale yellowish suspension was observed. The obtained NPs were centrifuged at 13,500 rpm for 10 min and separated from the supernatant. The precipitate was washed three times with distilled water and two times with ethanol to get rid of unreacted amine and other by-products. The products, ZnS(1) and ZnS(2), were dried in air and used for further characterization and tests.

### 2.4. Characterization of ZnS Nanoparticles

The dry powders of samples ZnS(1) and ZnS(2) were used for X-ray diffraction (XRD), X-ray photoelectron spectroscopy (XPS), scanning electron microscopy (SEM), Fourier transform infrared (FTIR) spectroscopy, and photocatalytic tests. They were redispersed in ethanol by means of sonication, after which they were drop-cast onto Si wafers for XRD, XPS, and SEM measurements, while more diluted dispersions were used for UV–Vis absorption spectroscopy.

The XRD patterns of the products were recorded on a Bruker instrument (D2 Phaser) with Cu/Ni radiation (λ = 0.154184 nm) at room temperature. UV–Vis absorption spectra were obtained on a BOECO S-220 spectrophotometer. Field-emission SEM (FE-SEM) images were taken on a Hitachi S-4800 microscope. The XPS measurements were carried out on an ESCAlab-MkII spectrometer (VG Scientific). The FTIR spectra of the samples in KBr tablets were recorded in the range 4000–400 cm^−1^ at a resolution of 1 cm^−1^ using a Nicolet 6700 FTIR spectrometer.

### 2.5. Photocatalytic Tests of ZnS Nanoparticles

The photocatalytic experiments were performed using 100 mL of aqueous methylene blue (MB) solution (with concentration 8 × 10^−6^ mol/L) and 10 mg of each catalyst according to previously described methodology [39]. Prior to irradiation with visible light, the mixture was stirred in the dark for 30 min to achieve adsorption–desorption equilibrium for each sample tested. The light source was a tungsten halogen lamp (power 1000 W) fixed at a distance of 40 cm above the beaker with the tested mixture. The temperature of the liquid was controlled at 25–26 °C by means of an ice bath. After each 30 min interval, 4 mL of the dispersion was taken, centrifuged, and analyzed by UV–Vis spectroscopy, after which the analyzed aliquot was redispersed by sonication and returned to the beaker for further reaction.

## 3. Results and Discussion

**Scheme 1:**
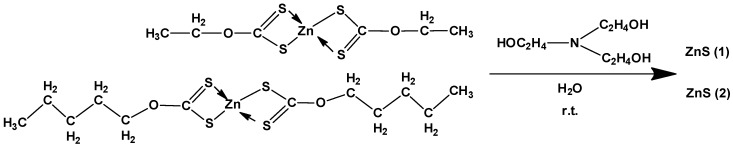
Reaction conditions for preparation of samples ZnS(1) and ZnS(2).

**Figure 1:**
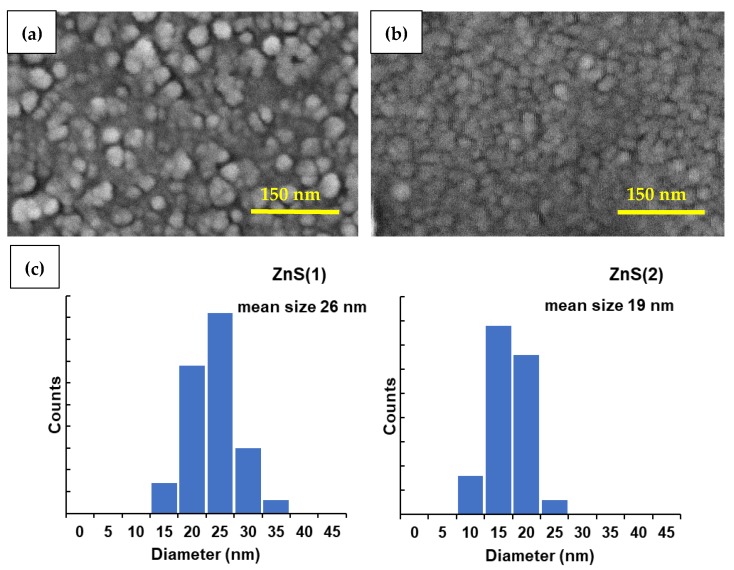
Field-emission scanning electron microscopy (FE-SEM) images of the prepared ZnS NPs: (**a**) sample ZnS(1) prepared using zinc ethylxanthate; (**b**) sample ZnS(2) prepared using zinc amylxanthate; (**c**) Histograms of particle size distribution for samples ZnS(1) and ZnS(2).

The reactions between metal xanthates and excess of tertiary amine were carried out for a prolonged time at room temperature. The NPs of samples ZnS(1) and ZnS(2) were formed by slow decomposition of zinc ethylxanthate and zinc amylxanthate, respectively, in the presence of triethanolamine, as shown in Scheme 1 below:

FE-SEM images of sample ZnS(1), prepared from zinc ethylxanthate, and sample ZnS(2), prepared from zinc amylxanthate, are presented in Figure 1 as panels (a) and (b), respectively. It can be seen that both samples had nearly monodispersed spherically shaped particles, with mean sizes of 26 nm for ZnS(1) and 19 nm for ZnS(2). The size distribution was within the narrow ranges of 15–40 nm and 10–30 nm for ZnS(1) and ZnS(2), respectively (see histograms in Figure 1c). 

The formation of uniform ZnS nanospheres could be ascribed to triethanolamine molecules, which acted as capping agent and prevented further aggregation of NPs that were forming. Triethanolamine is also believed to slow the release rate of S^2−^ ions coming from xanthate and contribute to controllable growth of ZnS nanocrystals. As a nucleophile, triethanolamine was able to attack the positively charged carbon center in xanthate, thereby causing cleavage of the C–S bond and elimination of the sulfide ion. Then, the sulfide ions generated in situ reacted with zinc ions, forming tiny nanocrystals of ZnS. As clearly seen in Figure 1, the ZnS NPs were smaller when zinc amylxanthate was used as a precursor. We suppose that kinetic factors were responsible for the observed size dependence. In the case of amylxanthate, the nucleophilic reaction was slower for two reasons: (i) the steric hindrance of the five-member-carbon chain in amylxanthate and (ii) the lower partial positive charge on the central carbon atom in amylxanthate owing to a greater inductive effect of its long-chain alkyl group. These two factors resulted in a slower release of sulfide ions from amylxanthate, which in turn limited the size of the growing ZnS NPs.

**Figure 2:**
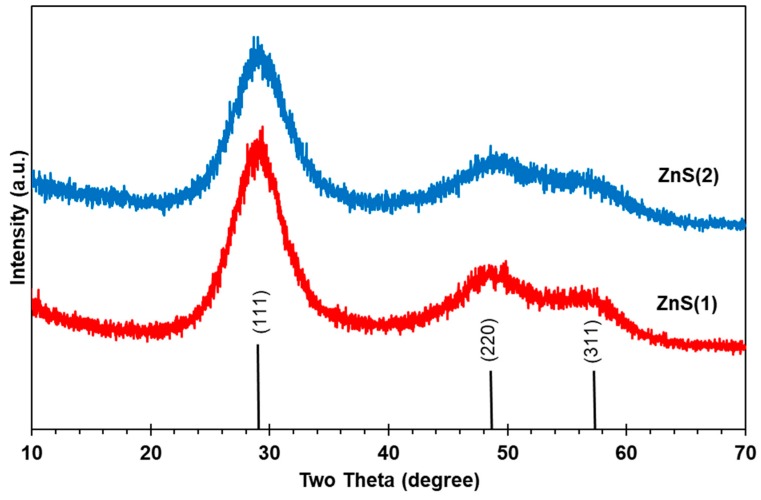
X-ray diffraction (XRD) patterns of samples ZnS(1) and ZnS(2).

The XRD patterns of samples ZnS(1) and ZnS(2) are shown in Figure 2, revealing single-phase ZnS materials. The three broad diffraction peaks observed in both patterns correspond to the crystallographic planes (111), (220), and (311) of cubic ZnS and are in agreement with the standard pattern (JCPDS card no. 05-0566). Using the Scherrer equation given below, crystallite sizes were calculated from the XRD patterns:

(1) $$L=\frac{0.9\;\lambda }{B\cos\theta }.$$

**Figure 3:**
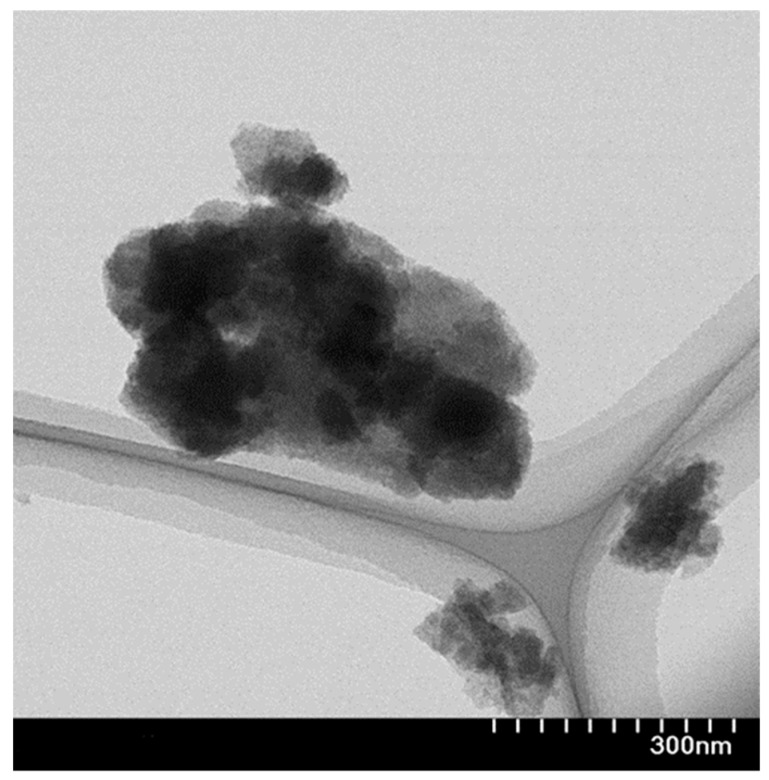
Transmission electron microscopy (TEM) image of sample ZnS(1).

In this formula, *L* is the coherence length, *λ* is the wavelength of the X-ray radiation used (0.15406 nm), *B* is the full width at half maximum (FWHM) of the most intense peak, and *θ* is the angle of diffraction. The relationship between the coherence length *L* and the diameter of a spherical NP (*D*) is known to be D=4L/3 [4,9]. The calculated crystallite diameters for samples ZnS(1) and ZnS(2) were 2.8 and 3.2 nm, respectively. Sample ZnS(2) had a slightly bigger crystallite size and showed a lower degree of crystallinity, as revealed by its broader peaks in the XRD pattern (see blue pattern in Figure 2) [1]. Owing to the very small dimensions and the high surface energy of ZnS crystallites in both samples, they easily aggregated to bigger polycrystalline NPs, as can be seen in the transmission electron microscopy (TEM) image of sample ZnS(1) in Figure 3. 

**Figure 4:**
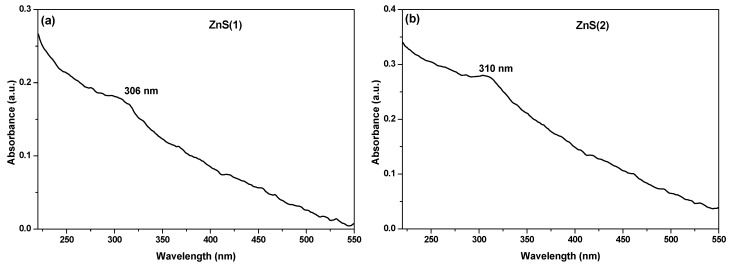
UV–Vis absorption spectra of samples (**a**) ZnS(1) and (**b**) ZnS(2).

The UV–Vis spectra of samples ZnS(1) and ZnS(2) dispersed in ethanol are shown in Figure 4. A broad long-wavelength absorption band was seen for both samples, alongside with distinct exciton absorption peaks observed at 306 and 310 nm for samples ZnS(1) and ZnS(2), respectively. The blue shift of these peaks in comparison with the absorption band at 336 nm for bulk ZnS material is attributed to the quantum confinement effect that is well known for nanosized particles [40,41]. Similar UV–Vis absorption spectra registered for 3 nm ZnS nanocrystals capped with polyvinyl alcohol (PVA), polyvinylpyrrolidone (PVP), and cetyltrimethylammonium bromide (CTAB) were recently presented in [10]. The band gap energy (*E*_g_) can be calculated using Plank’s equation, *E* = *hc*/*λ*, where *h* is the Plank constant, *c* is the speed of light, and *λ* is the wavelength at the absorption maximum [5,40]. Using this approach, the *E*_g_ values for samples ZnS(1) and ZnS(2) were found to be 4.05 and 4.00 eV, respectively, while bulk ZnS is known to have a band gap equal to 3.66 eV [28,42]. Generally, as a result of the quantum confinement effect, the band gap of semiconductor NPs increases with a decrease in NP size, which was indeed observed for both samples.

The elemental composition of the samples and oxidation states of atoms were determined by XPS analysis. Only Zn, S, C, O, and N atoms were found on the surface of the analyzed NPs, as revealed by XPS survey scans (not shown here). The signals for C and O arose not only from environmental contaminations but also from triethanolamine molecules capping the ZnS NPs. Table 1 shows the atomic percentage of all elements detected in the samples. As can be seen, both nanomaterials had somewhat larger atomic percentage of Zn than that of S atoms. This indicates nonstoichiometric composition and suggests formation of sulfur vacancies in the sample structures. The latter vacancies are expected to be responsible for enhanced photocatalytic activity of the samples, as discussed below. 

**Figure 5:**
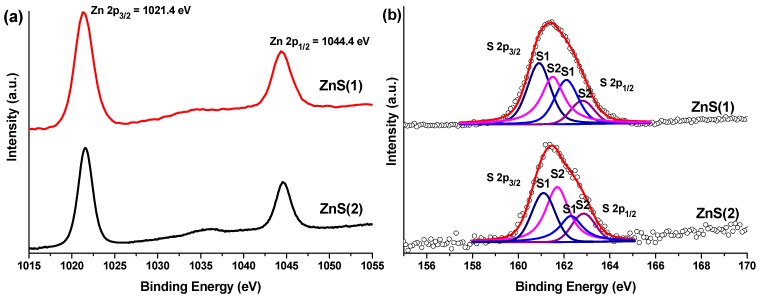
XPS spectra of samples ZnS(1) and ZnS(2): (**a**) Zn 2p and (**b**) S 2p.

Figure 5a,b presents the Zn 2p and S 2p XPS spectra of samples ZnS(1) and ZnS(2), respectively. The peaks at 1021.4 and 1044.4 eV were assigned to Zn 2p_3/2_ and Zn 2p_1/2_ levels and clearly indicated the presence of Zn^2+^ ions in the crystal lattice of both samples [43]. Liu and coauthors observed these peaks for ZnS NPs at 1022.1 and 1045.0 eV [25]. Wang et al. demonstrated low-energy shift of Zn 2p peaks with increasing amount of sulfur vacancies [3]. This allowed us to conclude that, for samples ZnS(1) and ZnS(2), the position of the Zn 2p peaks was also somehow affected by the presence of S vacancies. Further evidence for S defects can be gained from the S 2p XPS spectra shown on Figure 5b. As can be seen, the spectra were deconvoluted into four components, assigned to S 2p_3/2_ and S 2p_1/2_ levels from two sulfur species. One set was located at ~160.9 and ~162.1 eV and was assigned to S^2−^ ions (S 2p_3/2_ and S 2p_1/2_ levels) surrounded by four Zn ions in a regular tetrahedral geometry in the crystal lattice (denoted as S1 in Figure 5b). The second doublet of S 2p_3/2_ and S 2p_1/2_ appeared at ~161.5 and ~162.8 eV and was attributed to sulfur vacancies, more specifically to sulfide ions located in an irregular manner around the Zn ions in the structure of ZnS (denoted as S2 in Figure 5b). The higher binding energy values observed for S vacancies are in accordance with the trend for defect-rich ZnS nanocrystals previously reported in [3]. 

Additionally, the presence of organic molecules surrounding the ZnS NPs in both samples was confirmed by FT-IR spectroscopy. Table 2 lists the IR bands observed in the spectra of samples ZnS(1) and ZnS(2), along with their assignment, clearly indicating the presence of chemical bonds available in triethanolamine [44,45]. 

The photocatalytic activity of both samples was evaluated as they were dispersed in aqueous MB solution and irradiated with visible light. As an organic compound, MB has a stable aromatic structure and is often used as a model substance simulating organic pollutants in water. Its aqueous solution is known to show a strong absorption band at 664 nm with a shoulder at 610 nm, which are used for monitoring the decomposition of MB by photocatalytically active materials. The absorption spectra of MB solution in the presence of samples ZnS(1) and ZnS(2) were recorded after 0, 30, 60, 90, 120, 150, and 180 min under visible-light irradiation. The intensity of the absorption band was found to decrease gradually over time, indicating a steady degradation of the dye. The change in MB concentration (C/C_o_) and the degradation efficiency [(C_0_ − C)/C_0_ × 100, in %] were determined for both catalysts, which allowed us to plot the percentage of remaining MB within the irradiation time.

**Figure 6:**
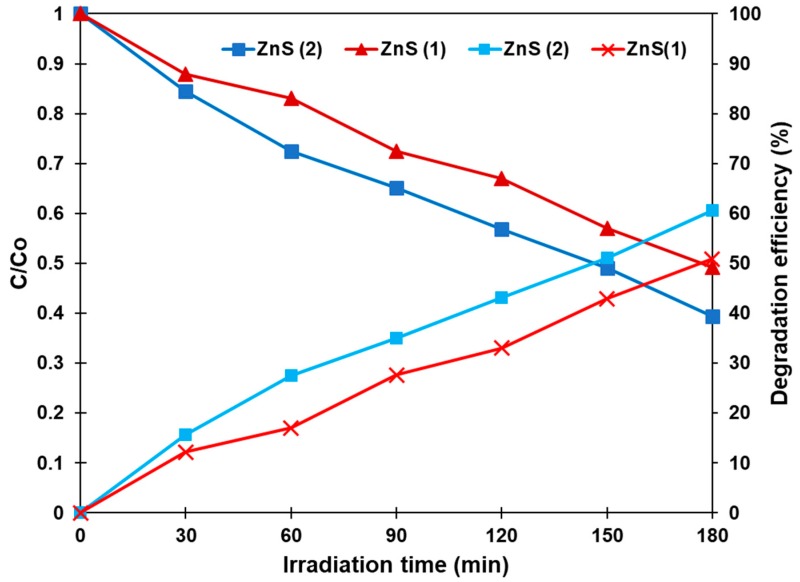
Methylene blue (MB) concentration change (C/C_0_) and degradation efficiency (%) over samples ZnS(1) and ZnS(2) during their irradiation for 180 min. Before illumination, each reaction mixture was kept in the dark for 30 min.

Figure 6 displays how the concentration of MB changed in the presence of both samples ZnS(1) and ZnS(2) for as long as 180 min. The degradation efficiency over this period of time was estimated as 51% and 60% for samples ZnS(1) and ZnS(2), respectively. The nanomaterial produced from xanthate with a longer carbon chain (sample ZnS(2)) exhibited somewhat higher photocatalytic activity compared with sample ZnS(1). This can be explained by its smaller particle size as well as the smaller value of band gap, with the combination of these two factors ensuring advanced photocatalytic performance. Remembering that the newly prepared nanomaterials were both deficient in sulfur, as shown by the XPS chemical analysis (Table 1), one can assume that the formation of sulfur vacancies in the ZnS crystal lattice should also contribute to an increase in visible-light absorption [38]. Previously, Wang et al. used density functional calculations to confirm that sulfur vacancies indeed generated new states located within the band gap (i.e., between the valence and conductive zones) of ZnS, which resulted in a decrease of band gap and enhanced visible-light absorption of ZnS [3]. Comparison between the areas of peaks S1 and S2 in samples ZnS(1) and ZnS(2), presented in Figure 5b, suggested that the concentration of S vacancies in sample ZnS(2) was greater than in sample ZnS(1). This is believed to be one of the reasons for the higher photocatalytic activity of sample ZnS(2). The stronger S2 component observed for ZnS(2) agreed well with the results given in Table 1, where it can be seen that the atomic ratio Zn:S was higher for sample ZnS(2) than for ZnS(1) (1.36 and 1.21, respectively), indicating that sample ZnS(2) was more sulfur-deficient.

On the other hand, such vacancies can serve as adsorption sites for oxygen molecules, which are reduced by photogenerated electrons in the conduction band (O_2_ + e^−^ → •O_2_), as well as adsorption sites for H_2_O molecules, where water undergoes oxidation by holes from the valence band (H_2_O + h^+^ → H^+^ + OH•). Thus, the presence of sulfur vacancies should prevent recombination of light-generated electrons and holes, which is a major factor for enhanced photocatalytic activity of the material. In addition, defects in the structure of the prepared nanomaterials may enhance adsorption of MB molecules on their surface and ensure better contact between MB and the oxidizing species that decomposed this organic dye. 

## 4. Conclusions

In this study, for the first time, facile synthesis of ZnS nanoparticles was achieved through the decomposition of zinc xanthates as molecular precursors in the presence of tertiary amine in aqueous solution and at room temperature. Such experimental conditions were shown to favor crystallization of ZnS in the cubic phase and formation of polycrystalline nanoparticles with sulfur defects on their surface. The particle size was found to be dependent on the carbon-chain length of the organic group in the xanthate: smaller nanoparticles (mean size 19 nm) were produced from the xanthate precursor with longer carbon chain, while larger particles (26 nm in diameter) were obtained from the xanthate with shorter carbon chain. Both ZnS samples showed blue shift of their exciton absorption band and higher energy band gap in comparison with bulk ZnS, which is in accordance with the quantum confinement effect. The newly prepared nanomaterials were tested as catalysts for photodegradation of methylene blue in aqueous solution under visible-light irradiation. Higher degradation efficiency was observed for the material with smaller particle sizes. This can be explained by the particle size, smaller band gap energy, and more surface sulfur vacancies, which enhanced the visible-light absorption, reduced the electron-hole recombination, and thus improved the photocatalytic performance of the material. 

## Figures and Tables

**Table 1:** Elemental analysis based on X-ray photoelectron spectroscopy (XPS) data for samples ZnS(1) and ZnS(2).

Sample	Peak	Position	Atomic %
ZnS(1)	C 1s N 1s O 1s S 2p Zn 2p	285.2 399.7 530.9 161.4 1021.4	28.18 3.65 11.53 25.66 30.98
ZnS(2)	C 1s N 1s O 1s S 2p Zn 2p	285.2 399.5 531.2 161.7 1021.6	34.56 2.82 14.62 20.38 27.63

**Table 2:** Infrared bands and vibrational modes assigned to samples ZnS(1) and ZnS(2).

**Peak position (cm^−1^)**	3430	2930	1640	1400	1130–1010
**Assignments**	ν_O–H_	ν_C–H_	δ_O-H_	δ_C–H_	ν_C–N_, ν_C–C_, ν_C–O_
